# The fully bio-based bilayered flame retardant treatment for paper via natural bio-materials

**DOI:** 10.3389/fchem.2024.1503694

**Published:** 2024-12-18

**Authors:** Zhihao Sun, Xinlong Liu, Qiaosheng Hu, Qing Zhao, Xuyao Qi, Fei Xu, Jingxian Li, Zhongfei Ma

**Affiliations:** ^1^ School of the Environment and Safety Engineering (School of the Emergency Management), Jiangsu University, Zhenjiang, China; ^2^ Key Laboratory of Gas and Fire Control for Coal Mines (China University of Mining and Technology), Ministry of Education, Xuzhou, China; ^3^ Lianshui People’s Hospital of Kangda College, Affiliated to Nanjing Medical University, Huaian, China; ^4^ College of Safety Science and Engineering, Nanjing Tech University, Nanjing, China

**Keywords:** natural bio-materials, self-assembly, bilayered structure, flame retardant treatment, wood-based paper

## Abstract

In this paper, we report a novel method for enhancing the flame retardancy of wood-based paper by utilizing natural biomaterials. The research constructed a bilayered structure coating on paper fiber surfaces, incorporating mixed starch (MS), adenosine triphosphate (ATP), and phytic acid (PA) as natural bio-based flame retardants. The structural configuration of the coating comprises MS/ATP and MS/PA, which were sequentially assembled as bottom and top parts, respectively, through pneumatic spraying. The surface morphological features and elemental distribution analysis of treated paper indicated that bio-materials were successfully assembled, which resulted in a uniform flame retardant coating on the paper fiber surface. Compared to the untreated paper, the limiting oxygen index of 20 bilayers (BL) treated paper increased substantially from 19.07% to 24.00%, and the thermogravimetric analysis showed out the residual char yield enhanced from 23.80% to 38.10% under nitrogen atmosphere. The cone calorimeter test results of 20 BL treated paper have approximately a 50.00% reduction than the untreated paper in both peak and total heat release rates. During thermal exposure, the top and bottom parts of the bilayered structure coating are successively flame retarded prior to paper fiber degrading. The method presented in this paper provides an environmentally sustainable approach for producing flame retardant wood-based paper.

## 1 Introduction

Paper, one of the most prominent natural plant fiber products, has served humans as a printing and packing material for millennia (Sakamoto and Okada, 2013). Nowadays, paper with advanced properties on breathable, flexible, economical, biocompatible, and harmless (Hui et al., 2018; Valls et al., 2019; Wang et al., 2020), which is still playing a pivotal role in numerous applications, such as writing (Chen C. et al., 2021), printing (Potter et al., 2019), packaging (Liu et al., 2017), medical applications (Chen et al., 2015), filtration processes (Cao et al., 2020), and more. The performance and characteristics of paper have significantly improved with the advancements in paper industrial processes (Thakur and Devi, 2022; Zhao et al., 2023), enabling its substitution for environmentally detrimental materials in specific applications (Li et al., 2019; Wei et al., 2024; Zhang et al., 2024). The inherent flammability of plant fiber presents a substantial potential safety risk on fire ignition and spread of paper and limits its application (Upadhyay et al., 2023). Thus, it is necessary to implement flame retardant treatment to improve paper fire safety and extend its usage (Zhang et al., 2020; Wang et al., 2023). In today’s materials safety research, eliminating material defects has become more and more important than before and has attracted the attention of many researchers (Fang et al., 2017; Kambli et al., 2018).

For flammable paper products, adding flame retardant agents represents a conventional approach to enhance flame resistance (Zhang, 2016; Zeng et al., 2023). The commonly used flame retardant agents in the paper industry and research mainly include organic compounds, inorganic compounds, or mixed ones, which are rich in nitrogen and phosphorus. In the paper production process, inorganic flame retardant agents like metal hydroxides, metal oxides, and boron/silicon components are often used as fillers for paper (Kim et al., 2018). When the paper is exposed to the fire, these fillers absorb the heat, and decomposite releases the noncombustible gases that dilute flame gases and air in the combustion zone. At the same time, the decomposite residues of fillers forms the protective inorganic layer on the paper surface (Hollingbery and Hull, 2010). However, these inner-added fillers of paper may also adversely affect the performance of final products (Wang et al., 2019).

Traditional chemical flame retardants, once widely used as halogenated agents, have been gradually found to generate harmful substances. As a result, they are now increasingly being replaced by more environmentally sustainable and effective agents, such as intumescent flame retardants (Jordanov et al., 2019; Tu et al., 2023). Intumescent flame retardants generate uniformly expanded carbon layers in the fire, which isolate the combustion heat transfer and inhibit the thermal decomposition of flammable substances (Tang et al., 2019). Intumescent flame retardants have been adopted increasingly in fire safety research for various flammable materials due to their environmental advantages and better performance than chemical flame retardants. Recently, the self-assembled method has given a new way to implement flame retardant treatment on many different materials. This method could conveniently and efficiently distribute flame retardants on the surface of materials while significantly reducing the impact compared to the inner-added method for thin materials like paper, fabrics, and films (Li et al., 2012; Liu et al., 2022; Zheng et al., 2022). Additionally, the self-assembled method allows for precise control, resulting in a formed coating that is usually ultra-thin (Qiu et al., 2018). Layer-by-layer assembly (LbL) is the classical self-assembled method that has been widely reported in previous studies for surface coating. In the LbL preparation preprocess, self-assembled materials are typically water-soluble, and the coating is assembled by immersing the surface alternately in different aqueous solutions. As a result, the assembled coating shows the interleaved stacking structure with different self-assembled materials. However, recent studies have mainly prepared the assembled flame retardant coating of thin materials with two different self-assembled. There is significant design potential for the structure and function of LbL coatings in thin materials to achieve superior flame retardant performance.

Biomaterials are commonplace biological materials that are more safe, sustainable, and economical than man-made materials. Biomaterials also provide a new opportunity for modern flame retardant research, which has faced a development bottleneck in human health and environmental protection (Hagenimana et al., 2005; Kaewtatip and Thongmee, 2012; Ren et al., 2020). There are increasing examples of biomaterials utilized in flame retardant studies. Starch is a common natural biomaterial utilized across various industrial sectors, including food, paper, and feed manufacturing (Park and Lim, 2019). It is noteworthy that starch may function as a primary carbon source for intumescent flame retardants due to its abundant hydroxyl groups (Chen et al., 2023). The research has demonstrated that cationic starch has enhanced the flame retardant performance of nanocomposites with starch-clay-TiO₂ (Rehman et al., 2021). Phytic acid, one of the phosphorus-riched organic acids from plants, also could satisfy the essential requirements of the acid source of intumescent flame retardants (Liu et al., 2020). The flame retardant research of textiles reported that phytic acid has reduced the flammability of polyester/cotton blend fabrics and improved material dripping-resistant properties simultaneously (Fang et al., 2021). This performance is also found in flame retardant paper, which is treated with modified phytic acid (Chen Q. J. et al., 2021). Adenosine triphosphate, another green flame retardant agent that has been used in plastic foam and cotton textiles by self-assembled, shows the efficiency performance of flame retardancy (Jeong et al., 2021; Song et al., 2023; Wang et al., 2024).

This study investigated the paper flame retardancy performance with fully bio-bassed bilayered surface coating, which is self-assembled by mixed starch, adenosine triphosphate, and phytic acid. These layers of the flame retardant coating were combined with electrical property differentials of materials in aqueous solutions. The bottom and top parts of the bilayered coating were designed with different thermal response temperatures to react successively when the paper was exposed to fire. The study conducted experimental evaluations of the flammability of treated paper and discussed the flame retardant mechanism from the perspective of intumescent flame retardants. Furthermore, the impacts of bilayered coating on mechanical properties, water absorption, and biodegradability of untreated and treated papers have been assessed.

## 2 Materials and methods

### 2.1 Materials

The bio-based materials, including starch, adenosine triphosphate, and phytic acid, utilized in the experiment were commercially obtained and were used as received. The paper was supplied by Shandong Chenming Paper Holdings Co., Ltd., and the specification was 70 g/cm³. The experiment employed two kinds of starch with a purity of 99%: the water-soluble starch and the cationic starch, which were provided respectively by Nantong Yunfeng Starch Co., Ltd. and Guangdong Hongxin Biotechnology Co., Ltd. The adenosine triphosphate (ATP) with a purity of 98% was purchased from Xi’an Hengji Chemical Co., Ltd. The phytic acid solution (PA) with a purity of 70% was purchased from Shandong Yousuo Chemical Technology Co., Ltd. All aqueous solutions in the study were prepared with self-made deionized water.

### 2.2 Bilayered deposition

**FIGURE 1:**
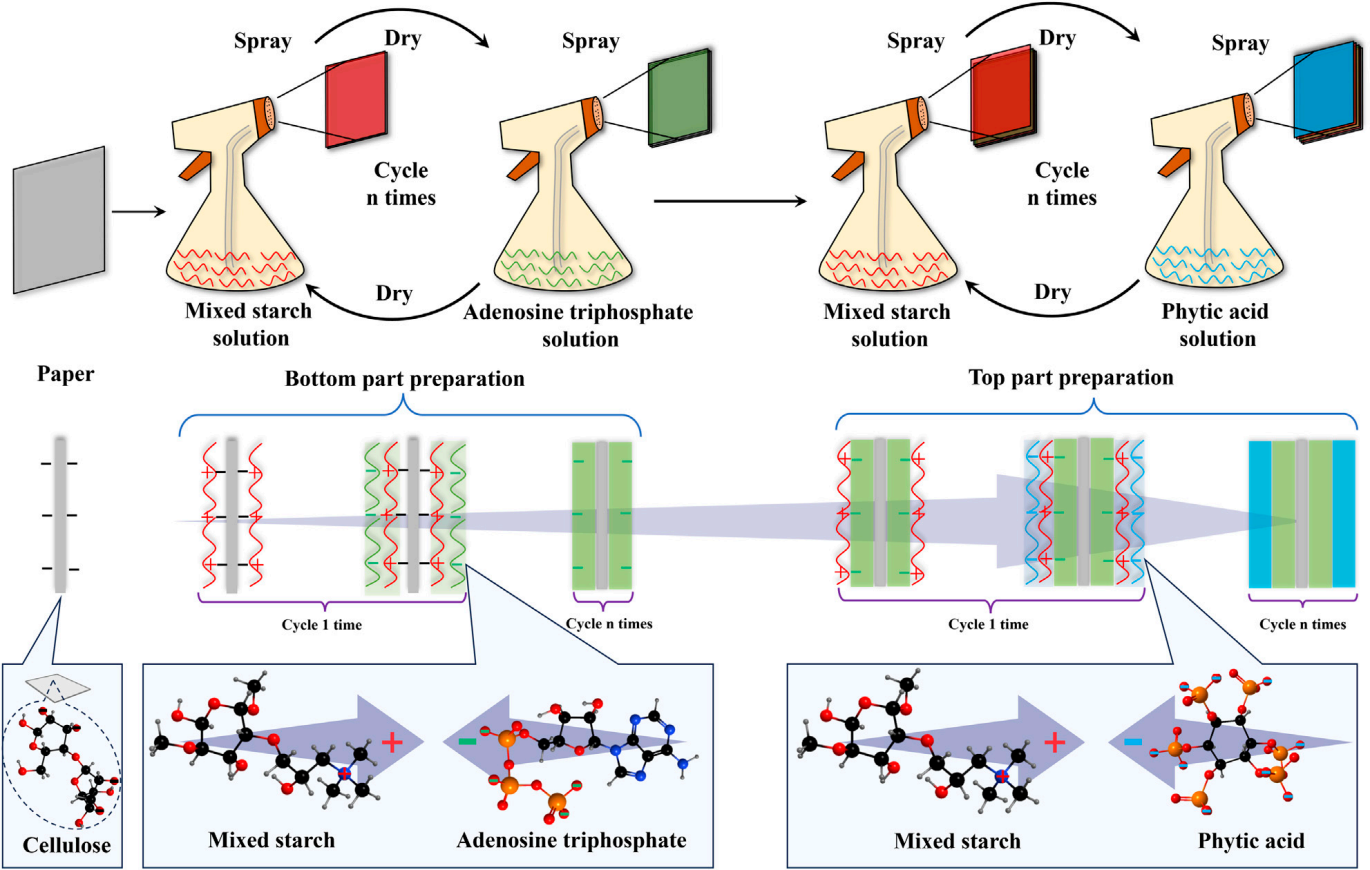
Schematic diagram of the flame retardant paper preparation process.

The water-soluble starch solution was prepared by dissolving water-soluble starch in deionized water with magnetic stirring at room temperature. The cationic starch was dissolved in deionized water with constant heating and stirring continuously until the powder was completely dissolved. The experiment utilized the same mixed starch solution (MS) for both the bottom and top part deposition of the bilayer coating. The MS is a mixture of water-soluble starch solution and cationic starch solution, which was mixed and homogenized under 90°C, then cooled at room temperature following well mixing. During the cooling process of the mixed solution, the intermolecular hydrogen bonds gradually formed, and the different starch molecules were connected. The mass concentration of MS prepared in the study is 2%, and the mass ratio between water-soluble starch and cationic starch is 19:1. The solution preparation process of ATP and PA is the same as that of water-soluble starch solution. The mass concentration of both ATP and PA solutions is 5%. The bilayered flame retardant coating, including the bottom and top parts, was sequentially constructed using the self-assembly method. Figure 1 presents the schematic of the flame retardant paper preparation process. Before the coating application, the paper samples were dimensioned into suitable size and cleaned by the airflow. The standardized pneumatic spray methodology was employed to mist each solution to the surface of all paper samples for layer deposition. After deposition of each layer, all paper samples should be cleaned and then dried at 60°C for 5 min for thorough dryness. According to the design of the bilayered structure, the bottom part of the flame retardant coating was made with alternating layers of MS and ATP solutions, while the top part was similarly made with MS and PA solutions. The paper sample is labeled n BL when it has deposited n layers of each solution for both the bottom and top parts. Following this methodology, the paper samples of 4 BL, 8 BL, 12 BL, 16 BL, and 20 BL were prepared.

### 2.3 Characterization

Morphological imaging and elemental analyzing of both the paper samples and their combustion residues were conducted utilizing the JSM-7800F scanning electron microscope equipped with energy-dispersive X-ray spectroscopy under vacuum-sprayed gold conditions (SEM-EDS, Japan Electron Optics Laboratory Co., Ltd., JPN). The horizontal burning test of paper samples was assessed using the CZF-3 horizontal and vertical combustion tester with the UL94 method as reference (HBT, Nanjing Jiangning District Analytical Instrument Co., Ltd., CHN). The limiting oxygen index required for the combustion of paper samples was determined by the JF-3 oxygen index tester (LOI, Nanjing Jiangning District Analytical Instrument Co., Ltd., CHN). The iConemini cone calorimeter performed the combustion behavior of paper samples under 30 KW/m^2^ irradiative heat flux (CONE, Fire Testing Technology Ltd., United Kingdom). The TG 209F3 analyzer carried out the thermogravimetric analysis of paper samples within a temperature range of 35°C–800°C at a heating rate of 10°C/min under nitrogen and air atmospheres (TGA, NETZSCH Scientific Instruments Trading Ltd., GER). The Nicolet IS5 fourier transform infrared spectrometer conducted the fourier transform infrared spectroscopic analysis to the coating of paper samples within the wave range of 4,000 to 500 cm^−1^ (FTIR, Thermo Fisher Scientific Inc., United States).

Considering the possible impacts of the coating, a series of tests about mechanical properties, including tensile strength, tearing resistance, and folding endurance, were executed to evaluate the changes between the untreat and treated paper. The tensile properties of paper samples were determined by the tensile index, which was measured by the YC-KZ-W2 tensile strength tester with the constant rate of elongation method (TS, Shandong Yicheng Equipment Co., LTD., CHN). The tear index was evaluated using the YC-SL-B tear strength tester with the Elmendorf method to perform the tearing resistance of paper samples (TR, Shandong Yicheng Equipment Co., LTD., CHN). The folding endurance of paper samples was measured by the YC-NZ-MIT MIT folding endurance tester (MIT, Shandong Yicheng Equipment Co., LTD., CHN).

Furthermore, water absorption of paper samples was determined by capillary rise, which was measured using the Klemm method in 10 min. The adhesion strength of the flame retardant coating was determined by the weight changes rate in the mutual friction test of paper samples with a contact pressure of 2.50 KPa. The biodegradation tests of paper samples were conducted in the natural soil and water environments, and the tests used the weight loss as the biodegradable performance index.

## 3 Results and discussion

### 3.1 Coating assembly process

In recent studies, ATP, the biological energy converter for various organismal biochemical processes, has been used as the green intumescent flame retardant via its extremely intumescent char formation. PA is another biosource acid widely used as an acid source in intumescent flame retardants for many materials. Starch consists of glucose synthesized by the green leaves during photosynthesis, which acts as the energy store in plants. Native starches are long-chain carbohydrates with limited industrial applications because of their inherent properties. Modified starch fulfills the flaws of native starch. With physical, chemical, enzymatic, or combination treatment, the native starches have been modified for improved physiochemical characteristics and functionalities. The water-soluble and cationic starch used in this study are separately modified by radiation and cationization treatments. Starch undergoes a transition process of gelatinization when hearted in water. The hydrogen bond of the crystalline region breaks, and the water molecule binds to the starch molecule hydroxyl group, finally leading to the structure of starch dissociated. Due to the richness of hydroxyl groups in each starch molecular structure, the hydrogen bonding water-soluble and cationic starch molecules easily during the starch retrogradation process. Then, the hybrid structure of mixed starch formed while the solution cooled down.

In this study, the intumescent flame retardant coating took full advantage of PA and ATP rich in the phosphorus and nitrogen elements, combined with the typical biodegradable natural carbohydrate mixed starch rich in carbon elements, to act as the surface flame retardant for the paper. Figure 1 illustrates the interaction process of ATP and PA with mixed starch in coating preparation. In a water solution, the ATP or PA molecules release hydrogen ions, and the molecule that leaves is electronegative, while the mixed starch is expressed electropositive due to its cationic groups. As the product of cellulose, paper inherits the electrical properties of cellulose and shows electronegative in water.

Therefore, the self-assembly method sequentially adsorped the oppositely charged materials onto a substrate through the electrostatic interactions of different materials. Considering the different thermal response designs, the coating was prepared into a bilayered structure with the MS and ATP as the bottom part and MS and PA as the top part.

**FIGURE 2:**
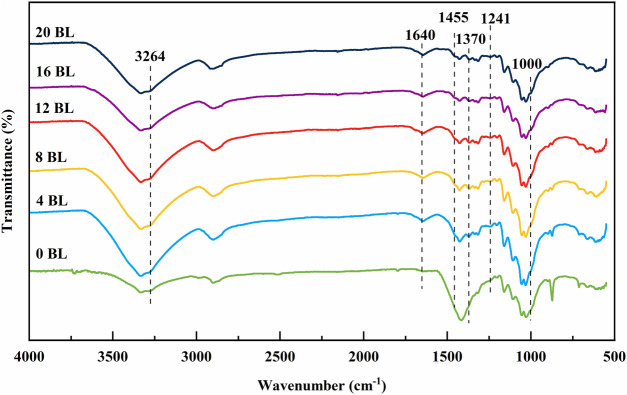
FTIR spectra of paper samples.

The paper samples were determined by Fourier transform infrared spectroscopy (FTIR). The results are shown in Figure 2. The absorption peaks at 1,640 cm^−1^ and 1,455 cm^−1^ are caused by stretching vibrations of the C = C and C = N bonds in ATP (Yin et al., 2023). The characteristic absorption peak of 3,264 m^−1^ is from ATP for the N-H bond (Cui et al., 2022; Wang et al., 2022). The CH_2_-O-CH_2_ stretching vibration band is 1,000 cm^−1^, which is the cationic amylose portion of the starch bonded in the starch backbone (Pal et al., 2005). The absorption peak at 1,370 cm^−1^ is the C-N stretching vibration peak of the quaternary ammonium group (Pal et al., 2005). The peak at 1,241 m^−1^ is from the vibration of COPO_3_, indicating the presence of PA (Sakai et al., 2017). In conclusion, the FTIR results show that the flame retardants were successfully bound to the paper.

### 3.2 Assembled coating characterization

**FIGURE 3:**
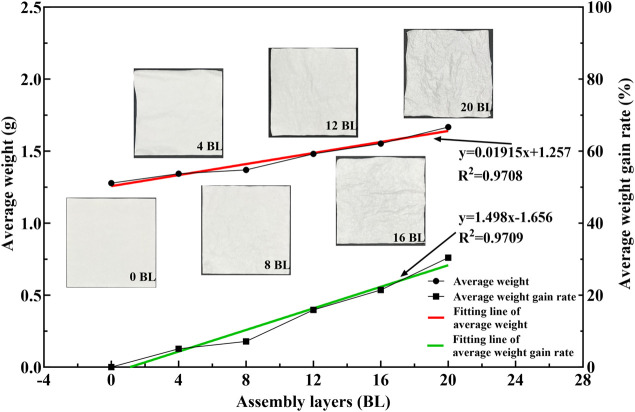
Weight variations and photographs of paper samples.

Both the average weight and its gain rate of paper samples are shown in Figure 3. The average weight and its gain rate of paper samples exhibit a consistently increasing trend by different samples; all the R-squared of each fitting line is above 0.97, indicating that the sample preparation process was reliable. Compared with untreated paper, the average weight of the 20 BL samples increased by nearly 30%, which was contributed by the assembled flame retardant coating on the paper surface. The variety of flame retardants helped reduce the flammability of the paper, but repeatedly using water solution treatment also made the paper more wrinkled. The photographs of the paper sample provided in Figure 3 clearly show that the paper appears wrinkled while the layers above 12 BL. The surface morphological images obtained by scanning electron microscopy provide visualization information on the microstructure of the coating.

**FIGURE 4:**
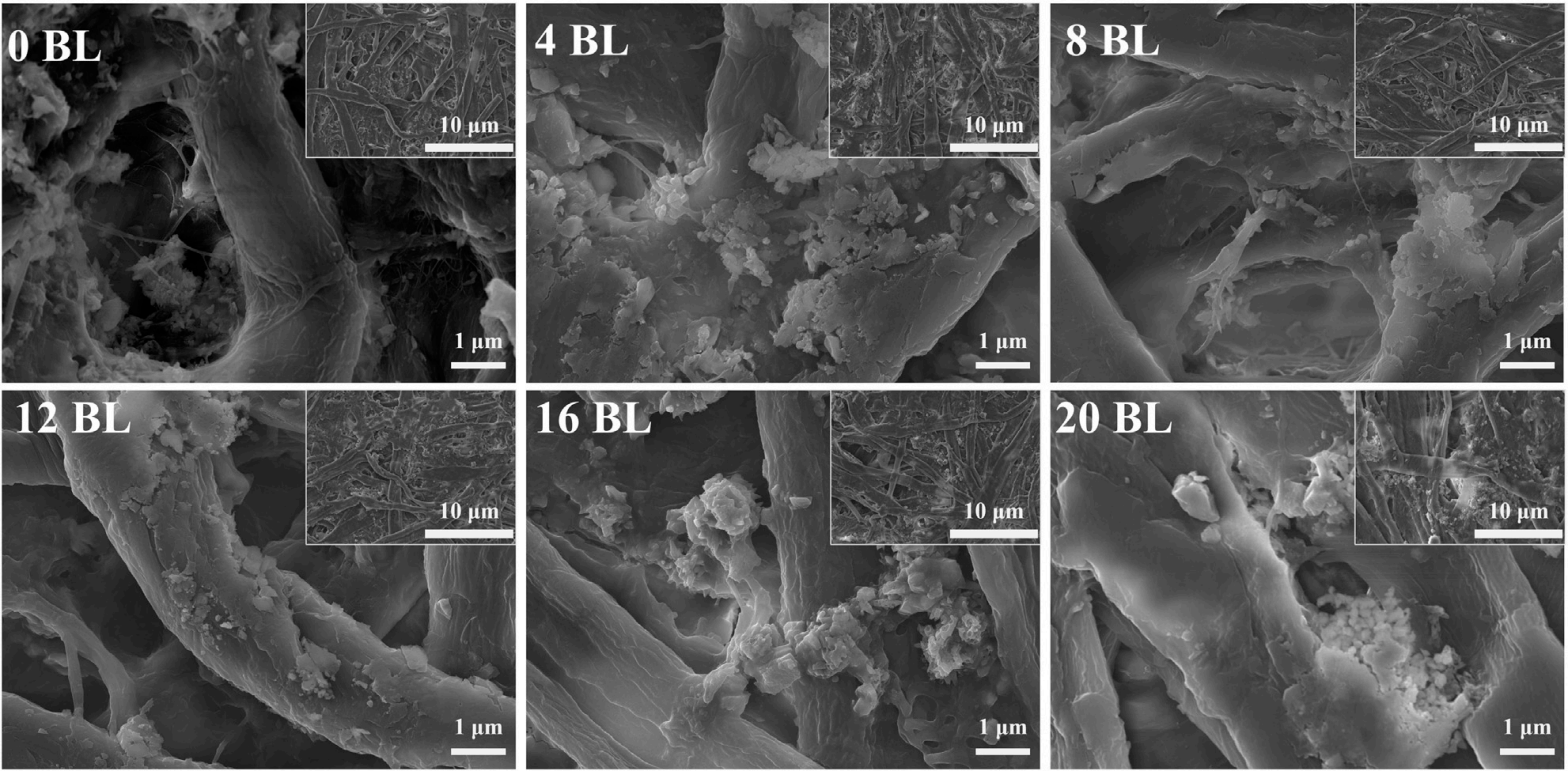
Microscopic morphology of paper samples.

**FIGURE 5:**
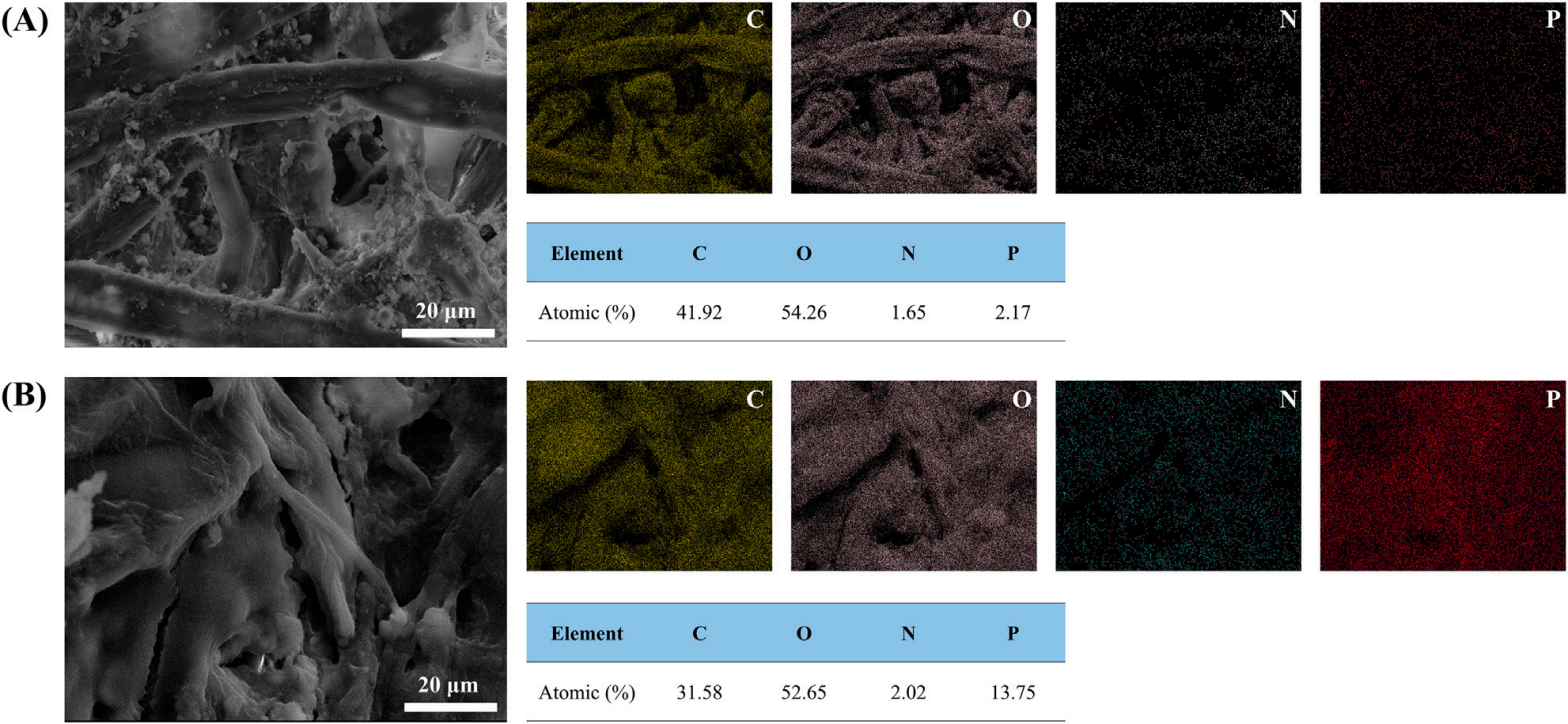
Elemental mapping of 0 BL and 20 BL paper samples.

Figures 4, 5 are imaged by SEM-EDS, in which Figure 4 gives the microscopic morphology of paper samples with different assembled layers, and Figure 5 shows the elemental mapping of 0 BL and 20 BL paper samples. The cellulose fiber in the untreated paper is clear, and its surface is smooth, while the fiber gaps are large and clean. With flame retardant constantly assembled, the cellulose fiber surface thickens and coarsens, revealing that the flame retardant successfully deposited and completely capsulated the fiber surface, providing complete fire protection to the fiber. Simultaneously, the flame retardant also acts interstitial the fiber gaps infill, sealing the superficial structure of the paper and forming a barrier, which may work like the firewall that helps impede the combustion heat and gases penetrating the inner space when the paper is exposed to the fire. These flame retardant effects will improve as the number of coating layers gradually increases, thus enhancing the flame retardancy of paper. Further, the elemental mapping shown in Figure 4 suggests that P element content has significantly increased in treat paper, which reflects lots of ATP and PA have been deposited on the paper surface. Meanwhile, the N element on the treated paper surface only slightly increases due to its lower relative content in cationic starch and ATP. With the increasing percent of P and N elements, the related percent of C and O elements reduced in varying degrees. The C and O elements shown in Figure 5 are not only from the flame retardants but also belong to the cellulose of paper fiber. Over the full scene of elemental mapping, the whole distributions of C, O, N, and P elements indicate that all the flame retardants have been distributed uniformly on the paper surface.

### 3.3 Flame retardancy of treated paper

**FIGURE 6:**
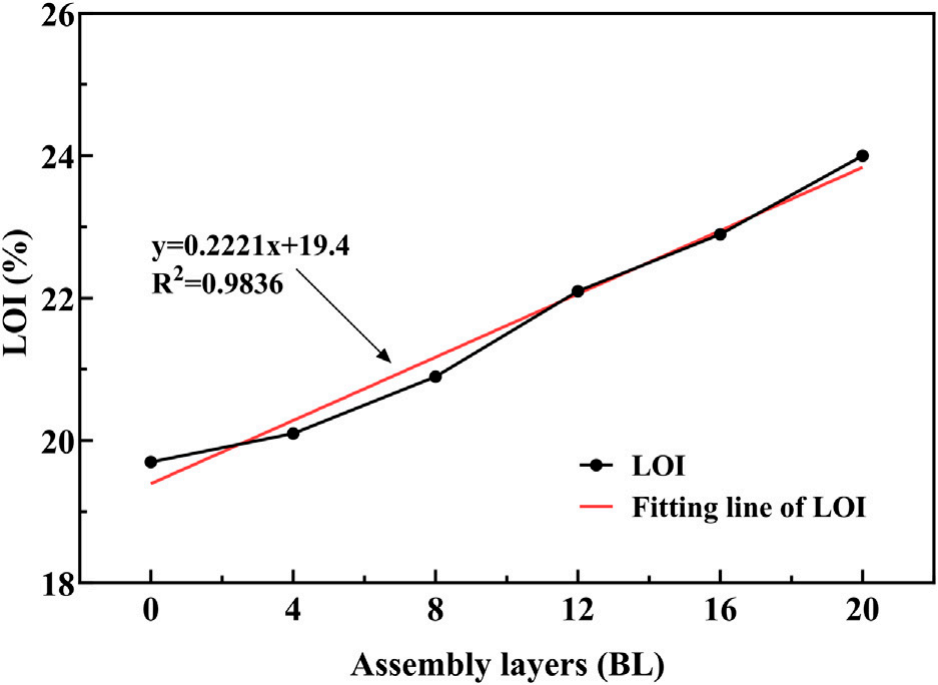
LOI of paper samples.

The limiting oxygen index (LOI) is the key factor that represents the flammability of the materials, it means that the minimum level of oxygen in the atmosphere is necessary for material combustion. Figure 6 gives the LOI changes with the flame retardant layers increasing in the paper samples. With paper assembled more and more cycles, the flame retardants persistent deposited on the paper surface, and made the LOI improve directly. Based on the LOI of 19.70% for untreated paper, the LOI performed better linear growth as 20.10%, 20.90%, 22.10%, 22.90%, and 24.00% for 4 BL, 8 BL, 12 BL, 16 BL, and 20 BL samples, in which R-squared of the fitting line is 0.9836. The linear growth reflects those flame retardants deposited homogeneously in each layer, and the flame retardancy of the treated paper is regulatable through the cycled times in the preparation process.

**FIGURE 7:**
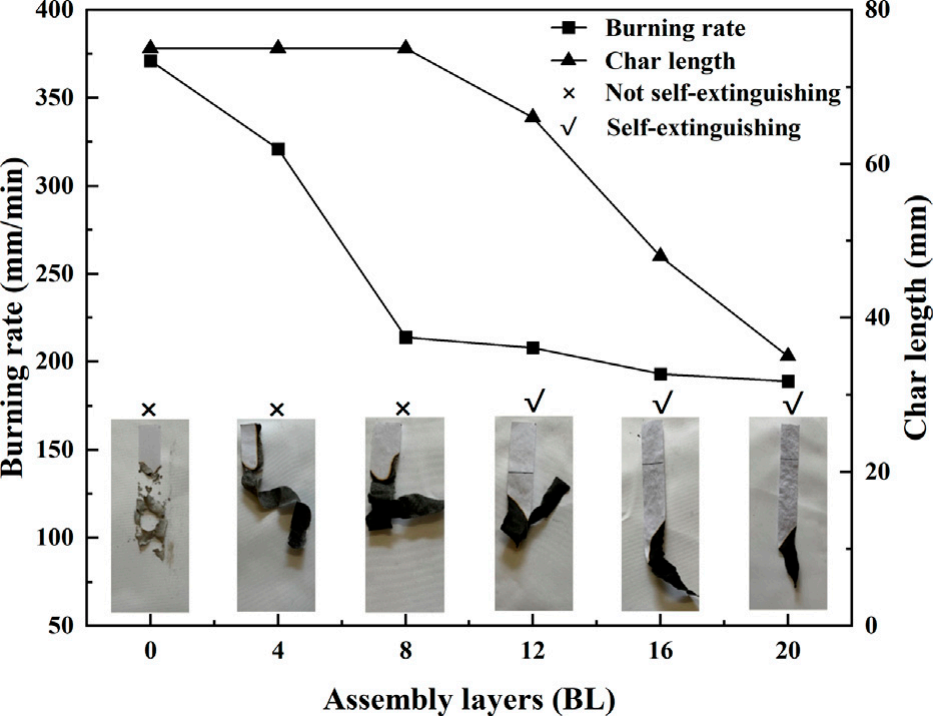
The HBT results and photographs of paper samples.

Furthermore, the horizontal burning test (HBT) is carried out to explain in more detail the flammability exhibition of the treated paper and the results are shown in Figure 7. The horizontal burning test intuitively revealed the ability of the test materials to extinguish or spread the flame after ignition, and it is intended to determine the linear burning rate of materials under specific test conditions. The burning rate indicated that the flame spread ability decreased with the flame retardant layers increasing in the paper samples in the HBT, in which samples have self-extinguished while the coating above the 12 BL. In Figure 7, the burning rates of 0 BL, 4 BL, and 8 BL samples show a nearly linear descent, in contrast to the 8 BL, 12 BL, 16 BL, and 20 BL samples are almost the same about 200 mm/min. Meanwhile, the 0 BL, 4 BL, and 8 BL samples are all burning down in the test, thus the residual length of the 8 BL, 12 BL, 16 BL, and 20 BL samples present is gradually growing. Both burning rate and residual length indicated that the flame retardants could quickly slow down the flame spread rate of paper samples to a limiting value, then the more flame retardants could not decrease the flame spread rate anymore, but they contributed to the earlier self-extinguishing time.

The photographs of the tested paper samples in HBT, which are given in Figure 7, show the different appearances of the burned residues. The burned residues of the untreated paper appears black-gray, while the color progressively turns black with the flame retardants coated. The change in the burned residues color may be caused by carbon layers produced in the intumescent flame retardant process.

**FIGURE 8:**
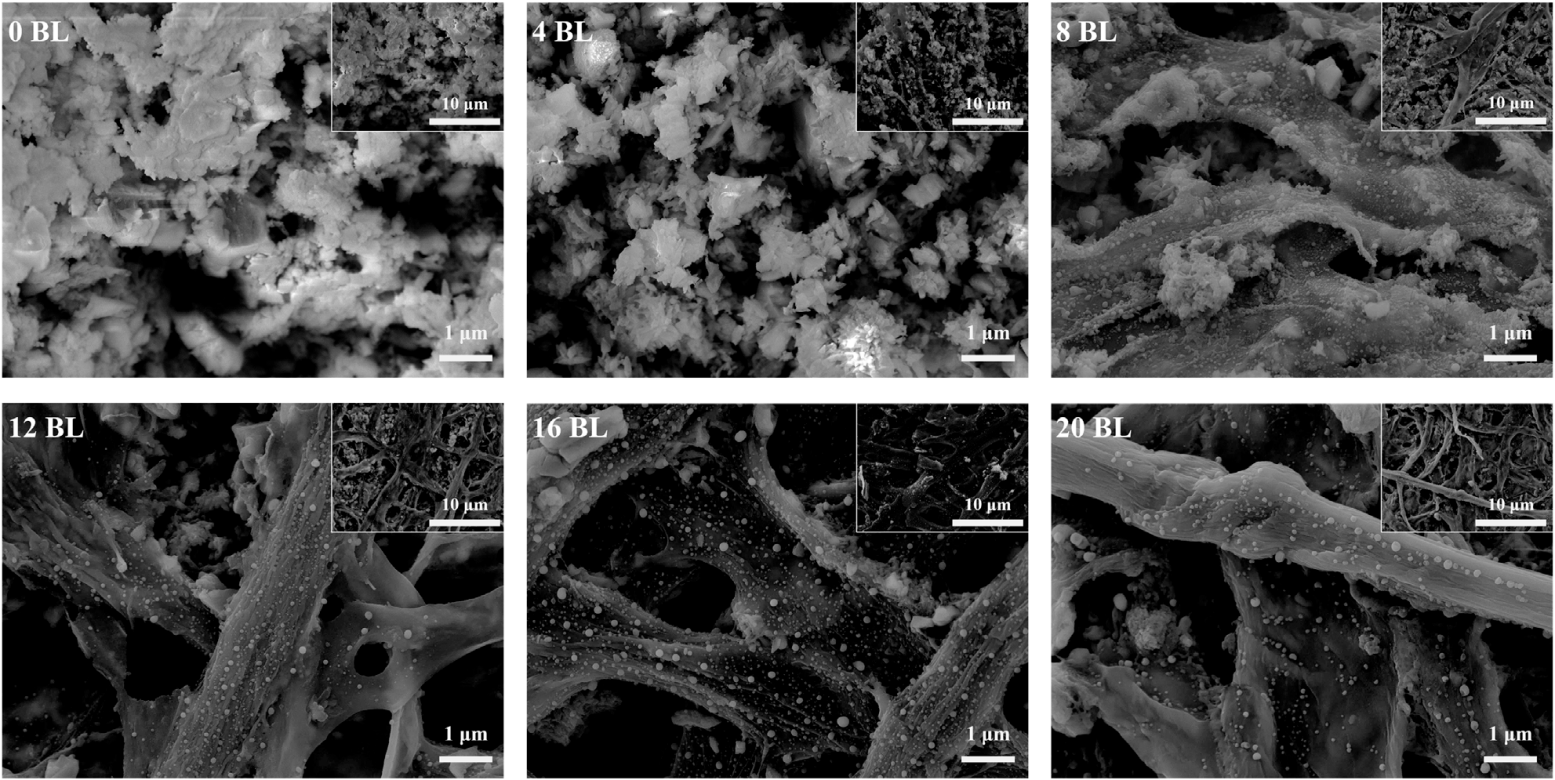
Microscopic morphology of paper sample burned residues.

**FIGURE 9:**
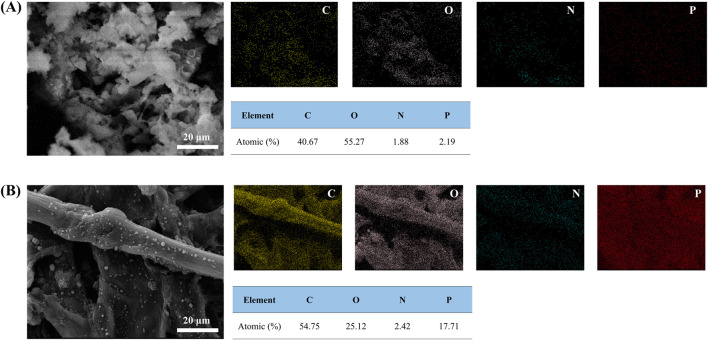
Elemental mapping of 0 BL and 20 BL paper sample burned residues.

The microscopic morphology and elemental mapping of the paper sample burned residues post-HBT have been imaged by SEM-EDS to acquire further information about how flame retardants work. Figure 8 exhibits the microscopic morphology of paper sample burned residues, and Figure 9 shows the elemental mapping of 0 BL and 20 BL paper sample burned residues. The burned paper fiber with no flame retardant is too loose to provide structural support for the burning residues of paper post-HBT. So the burning residues of the untreated paper appeared fragmentally, which is similar to the macro performance of the paper burning residues. However, the burning residues of paper samples with flame retardants maintained complete fiber morphology with dense residue structure characteristics, and this performance is greatly enhanced with the increase in the coating layers. The vision and texture comparison of paper fiber among paper samples demonstrates that the flame retardant offers effective fire protection, that the treated paper fiber exhibits less carbonation shrinkage, and is covered with a thick intumescent carbon layer.

Meanwhile, there are many closed bubbles appeared on the surface of the treated paper fiber, suggesting the quantity of inert gases, including CO_2_, H_2_O, and NH_3_, generated during the intumescent process of the flame retardant coating (Fang et al., 2021). These inert gases not only have the gas-phase flame retardant effect on the smothering but also play a bubbling role while overflowing the carbon layer. Then, the intumescent carbon layer will work as an insulating foam that isolates the heat and mass transfer processes between flame and paper.

The distribution of the C, O, N, and P elements shows different percentage changes in both the burned residues of the paper sample and the paper sample itself between pre-HBT and post-HBT. In the comparison of 20 BL paper burning residues to others, the C element content increased greatly, and a significant amount of C element is observed on the surface of burning residues, particularly on the paper fiber. And simultaneously where the O element content has substantially reduced, only less than half to the untreated one, declared fewer oxides stay on the surface of burned residues. Both enhancement of C element retention and reduction of O element content indicate the carbon layer formed on the paper surface, which will inhibit the combustion process and protect the paper fiber from combustion. The N element content left in 20 BL paper burning residues shows a smaller increase, while the P element has highly increased. The N and P elements of the treated paper are mainly from the flame retardants and play as the key electric connectors to assembled layers. The performance of both N and P elements represents that much of the flame retardants remain on the surface of burning residues, also reflecting that the carbon layer is transformed from the flame retardant coating.

**FIGURE 10:**
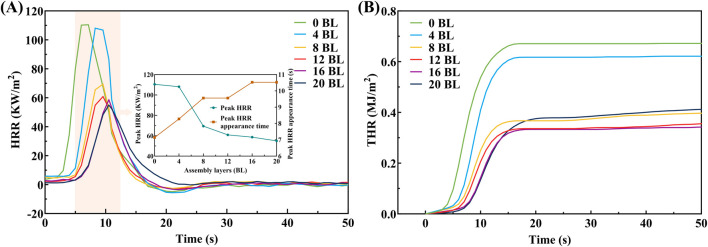
HRR **(A)** and THR **(B)** curves of paper samples.

The CONE and TGA are conducted to reveal the detailed process that how the flame retardant coating is working. The heat release performance of paper samples, through heat release rate (HRR) and total heat release rate (THR) measurements, is exhibited in Figure 10. The HRR curves exhibit progressive flat with coating layers increasing, in which peak HRR values gradually decrease and the appearance times are slightly lag. The peak HRR of 20 BL paper is 54.94 kW/m^2^ about 49.74% of the untreated paper, and its appearance time is correspondingly delayed by 3.39 s about 47.48% of the untreated paper. The changes in HRR show that the flame retardant coating notably inhibits the HRR of paper, and this effect increases with additional coating layers. This also reflects that flame retardant coating is transformed into the carbon layer covering the paper fiber surface, which disrupts fuel supply to the paper combustion. At the same time, the THR curve of paper samples shows that treated paper released less heat than the untreated one during the test, and the increasing rate of HRR also has a similar performance. The untreated paper has the highest final value of the THR at 0.67 MJ/m^2^, followed by the treated paper of 4 BL, 8 BL, 12 BL, 16 BL, and 20 BL. Although all bio-materials used in flame retardant coatings are flammable, the treated paper still exhibits a lower final value of the THR. This result reflects that flame retardants act earlier than paper combustion, and generate the carbon layer that protects the paper from combustion.

**FIGURE 11:**
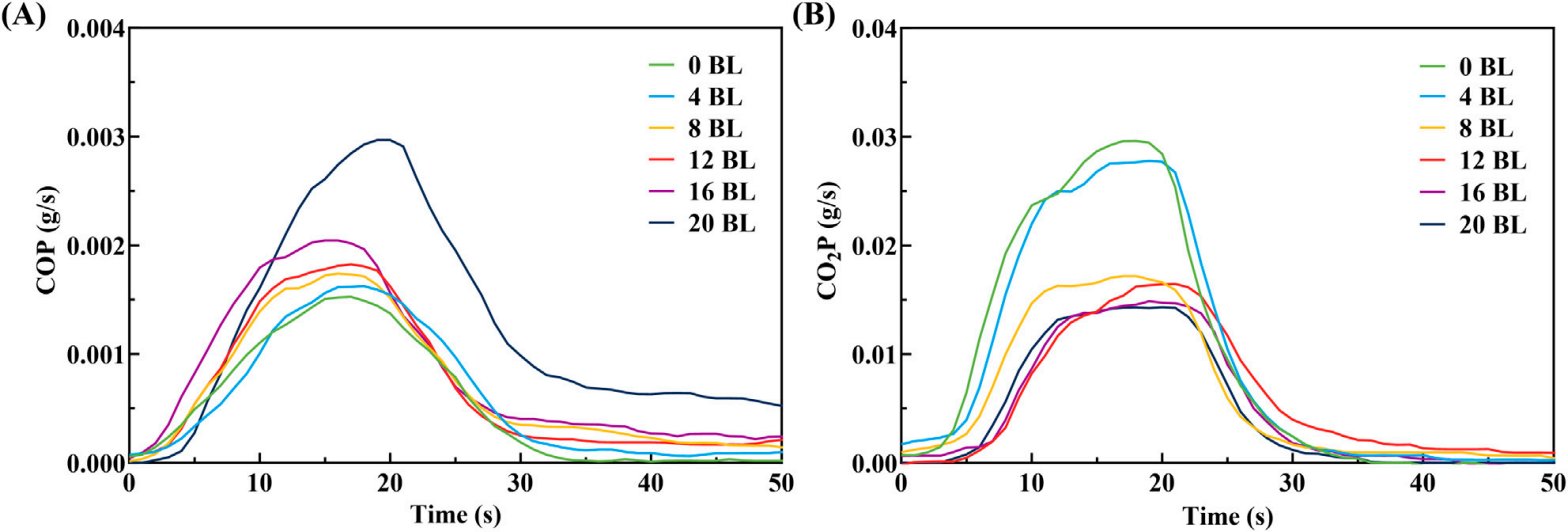
COP **(A)** and CO_2_P **(B)** curves of paper samples.

**FIGURE 12:**
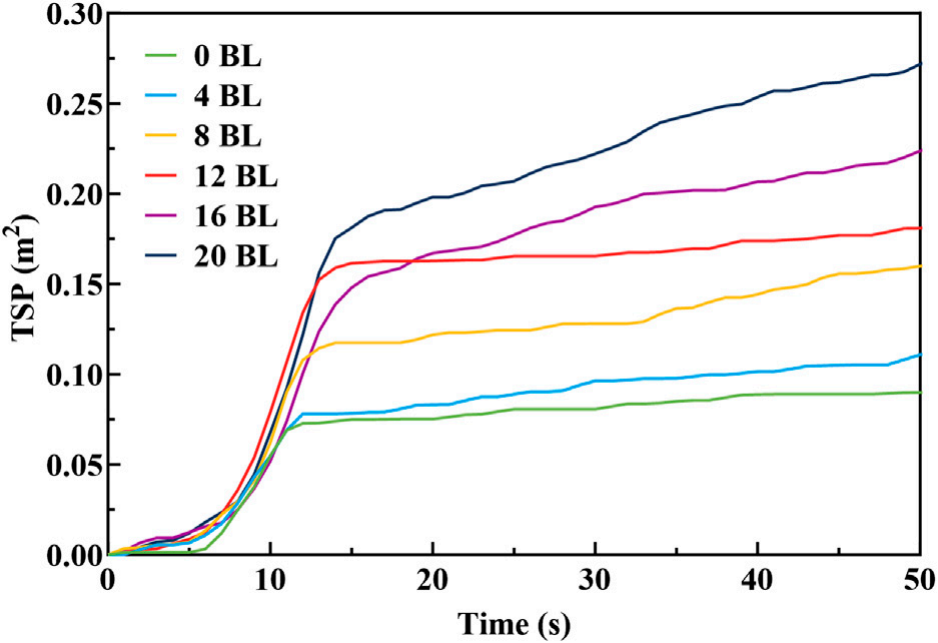
TSP curve of paper samples.

CO and CO_2_ are common gaseous combustion products, whose production rates directly report the combustion conditions. The CO and CO_2_ production rates (COP and CO_2_P) of the paper sample combustion processes have been synchronously measured in the CONE test, and the results are shown in Figure 11. The COP and CO2P exhibit opposite changes with the coating layers increasing: the COP is improved while CO_2_P is reduced. The untreated paper shows the lowest COP and the highest CO_2_P performance during combustion, indicating that the paper combusts completely. The flame retardancy of treated paper improves progressively with the coating layers addition, leading the combustion shift from complete to incomplete which is performed as COP increases and CO_2_P decreases in the test. The 20 BL paper exhibits the highest COP peak value for 0.0030 g/s among the treated paper samples, while the others perform nearly similarly in the range from 0.0016 to 0.0020 g/s. At the same time, The CO_2_P peak value of 4 BL paper is 0.0278 g/s, significantly higher than the others which range from 0.0172 to 0.0143 g/s. The total smoke production (TSP) of paper samples, shown in Figure 12, shows that treated paper produced more smoke in the test. The smoke released primarily comes from the incomplete combustion of the flame retardant coating on the paper samples. The variation in smoke production indicates that incomplete combustion is intensified with the coating layers increasing. The incomplete combustion carbonizes flame retardant coating to form a protective carbon layer. These phenomenons suggest that treated paper, which is coated with more flame retardants with increasing layers, will generate more char residues during incomplete combustion and transform into a stronger carbon layer.

**FIGURE 13:**
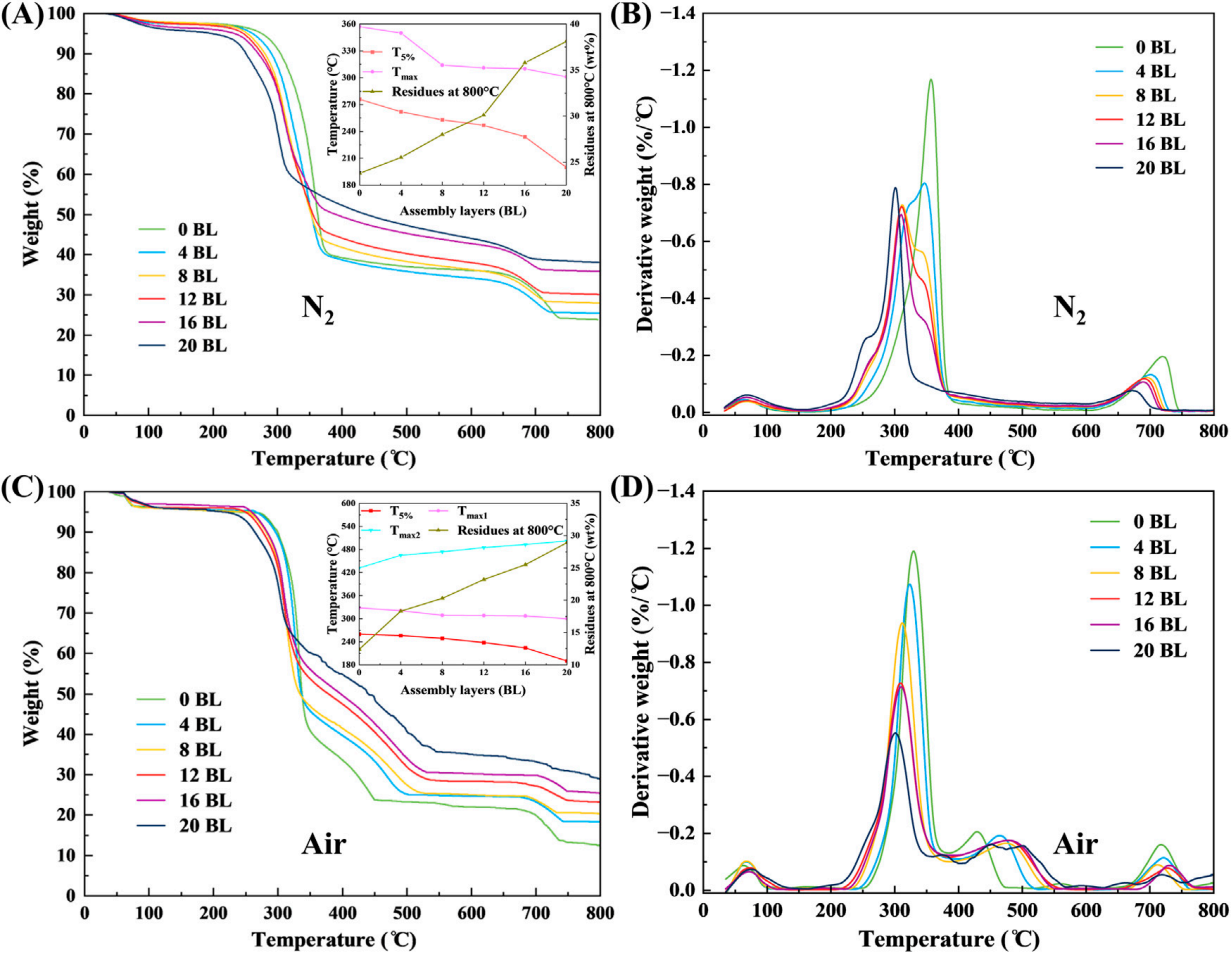
TGA **(A)** and DTG **(B)** curves of paper samples under nitrogen atmosphere, TGA **(C)** and DTG **(D)** curves of paper samples under air atmosphere.

TGA exhibits direct information about thermal decomposition through the thermogravimetric variation of paper samples under the nitrogen and air atmospheres, and the test results are shown in Figure 13. The 5 wt% weight loss temperature (T_5%_) is recorded as the initial temperature of thermal decomposition, and the T_max_ represents the temperature of the maximum decomposition rate. All the paper samples show a one-step decomposition under the nitrogen atmosphere with a temperature range of 200°C–400°C. The decomposition process of the untreated paper begins at 276°C with the T_max_ occurring at 357°C, while these temperatures appear lower for the treated ones. The decreasing trend of T_5%_ and T_max_ follows the addition of coating layers. When the coating layers reached 20 BL, the T_5%_ and T_max_ respectively reduced to 200°C and 301°C, indicating the responding temperature of coating is much lower than that of the paper thermal decomposition. This is probably caused by the PA, whose pyrolysis temperature is usually about 180°C–220°C and is used in the top part of flame retardant coating. With the temperature increasing, the flame retardant coating continuously carbonizes and generates the char residues to form the carbon layer, which could protect the paper from fire. This is also represented by the weights of residues in TGA. The main component of the paper samples residues in the test is char residues under the nitrogen atmosphere. As shown in Figure 13, the weight of paper sample residues shows the significance increasing with coating layers addition. The weight of the 20 BL paper residues is 38.10%, which is 1.60 times of the untreated paper (23.80%). The thermal oxidation of paper shown in TGA under the air atmosphere, is performed as a two-step process. The paper samples show a similarly decreasing trend in thermogravimetric variation. The untreated paper has the highest initial temperature of thermal decomposition, followed by the treated paper whose layers increase. The temperature difference between untreated paper and 20 BL paper is 70°C. With the temperature growing up, the first step of decomposition of paper samples mainly occurs at 210°C–350°C, which involves depolymerization and dehydration to form volatile products and aliphatic char (Wang et al., 2024). Then the second step is followed up at 300°C–550°C, which indicates some aliphatic char converted to aromatic char and others converted to volatile products under the action of hot oxygen (Wang et al., 2024). The temperatures of the maximum decomposition rate in two steps are respectively noted as T_max1_ and T_max2_. The T_max1_ variation of the paper samples is similar to the T_max_ which tested under the nitrogen atmosphere. This may also be caused by PA in the top part of the flame retardant coating. The T_max2_ shows increased trends between untreated paper and treated paper with different layers, which indicates the flame retardant coating has good protection and is enhanced as layers increase. The residues both of untreated and treated papers are performed similarly to those tested under the nitrogen atmosphere.

### 3.4 Mechanical properties and water absorption of treated paper

The mechanical properties of the paper samples are obtained through mechanical tests, with results in Table 1. Table 1 suggests that paper samples show an increasing trend in the tensile and tear indexes as the number of coating layers gradually increases, while the folding endurance attenuates slowly. Due to the adhesion effect of the flame retardant, such as MS, the cellulose fiber has been bonded tightly after the flame retardant fills the fiber gaps. So the tensile index of treated paper exhibits better performance compared to the untreated paper. Additionally, the strong binding strength of cellulose fiber contributes slightly to the tear resistance of paper, resulting in a minor improvement in the tear index. The flame retardant is mainly deposited and cured on the paper surface, which cannot provide the folding resistance effect. Contrarily, the cured flame retardant, similar to the hard particles with less elasticity, accelerates the damaging effect on the cellulose fiber during the folding process.

**TABLE 1:** Mechanical properties of paper samples.

Paper sample	Tensile index/N·m·g^−1^	Tear index/mN·m^2^·g^−1^	Folding endurance/times
0 BL	27.53	0.60	16
4 BL	33.69	0.64	15
8 BL	35.05	0.64	10
12 BL	36.94	0.64	9
16 BL	35.09	0.66	5
20 BL	39.10	0.68	4

**FIGURE 14:**
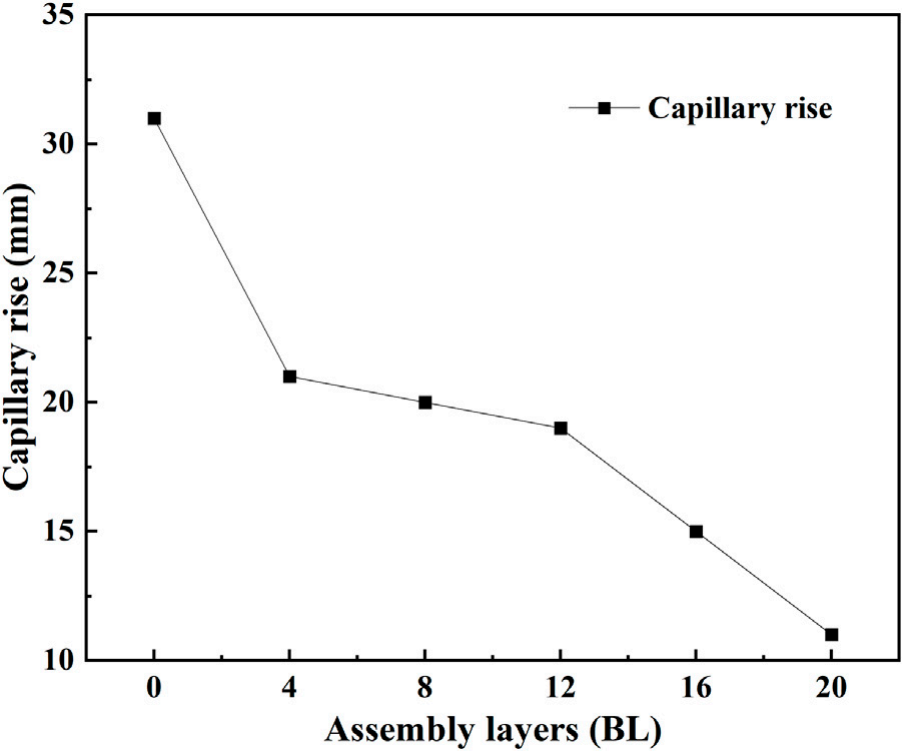
Capillary rise results of paper samples.

The water absorption of paper samples is tested following the Klemm method and evaluated by capillary rise. Figure 14 gives the capillary rise results of paper samples. Following the increase of the coating layers, the capillary rise results of paper samples show a quick decrease. According to the deposition situation of flame retardant on the paper surface, the fiber gaps have been filled by sediment deposits, and become more narrow or even disappeared. This will directly impact the capillary action of paper samples, and reduce their water absorbency ability.

The mutual friction of paper samples is carried under the fixed contact pressure, and the test results are listed in Table 2. After 500 times mutual friction, all the paper samples performed the good adhesion strength of the flame retardant coating, and only the 16 BL and 20 BL paper samples had slight weight loss which is less than 1%. For the 16 BL and 20 BL paper samples, the flame retardant coating is excessively thick, causing the flame retardant on the coating surface too easy to shed off. For the other paper samples, the flame retardant is mainly deposited on the fiber surface or fiber gaps, so the coating strength will be enhanced by the combined structure of the coating and fiber, just like the reinforced concrete structure.

**TABLE 2:** Mutual friction results of paper samples.

Mutual friction/times	Weight changes rate/%					
	0 BL	4 BL	8 BL	12 BL	16 BL	20 BL
0	100	100	100	100	100	100
50	100	100	100	100	100	100
100	100	100	100	100	100	100
200	100	100	100	100	99.8	99.7
300	100	100	100	100	99.7	99.6
400	100	100	100	100	99.6	99.5
500	100	100	100	100	99.4	99.3

**FIGURE 15:**
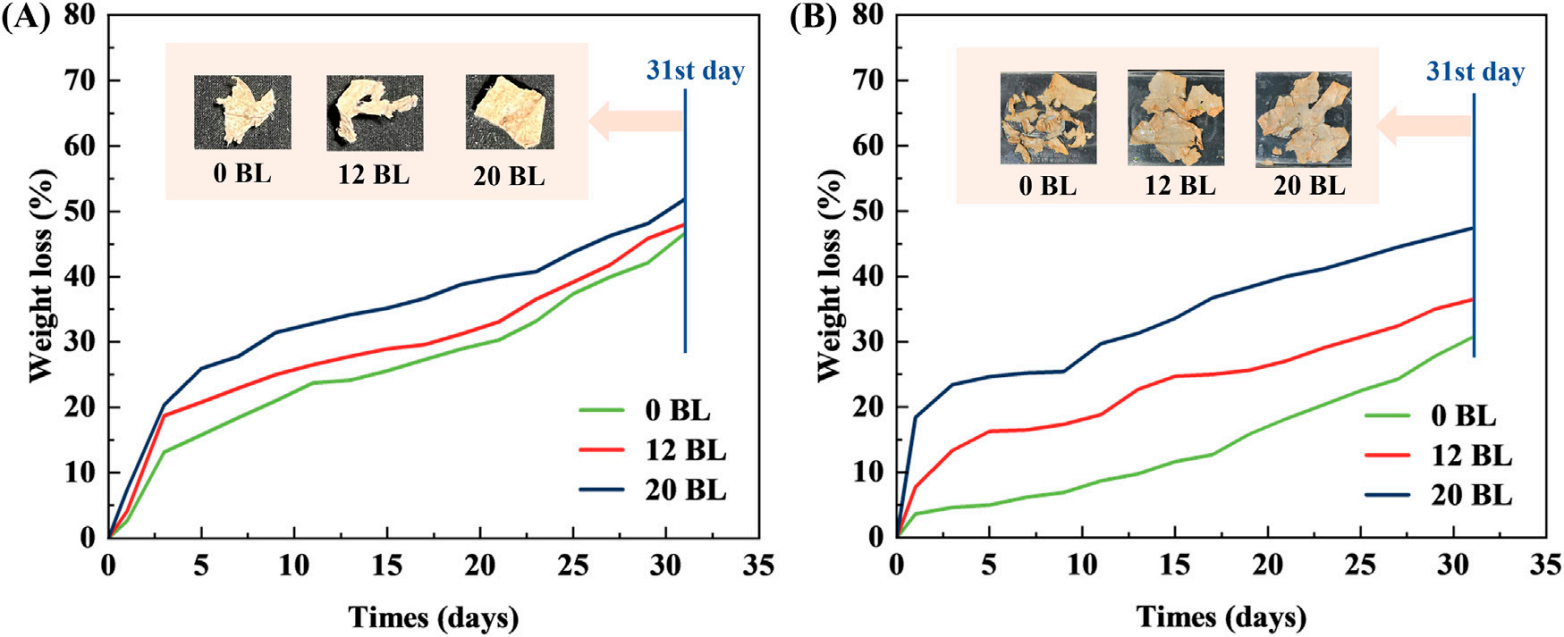
Weight loss of paper samples in natural soil **(A)** and water **(B)** environments.

The natural soil and water environments are used for the natural biodegradation of paper samples. The weight loss variations during 31 days and photographs of paper samples on the 31st day are both shown in Figure 15. All the paper samples exhibit the same biodegradation trends in soil and water environments according to the comparison between treated and untreated paper. All the weight loss curves indicate that treated paper biodegrades quicker than untreated paper. This phenomenon became more observable as the layers increased.

Meanwhile, the photographs of paper samples on the 31st day show that all the treated and untreated paper become tattered, reflecting the biodegradation process is effective. The better biodegradation performance of treated paper is mainly caused by the flame retardant coated on its surface. The components of flame retardant, MS, ATP, and PA, are all water-soluble biomaterials, which will easily dissolve into the environmental water and become nutrients for microorganisms in the natural environment.

### 3.5 Flame retardant mechanism

**FIGURE 16:**
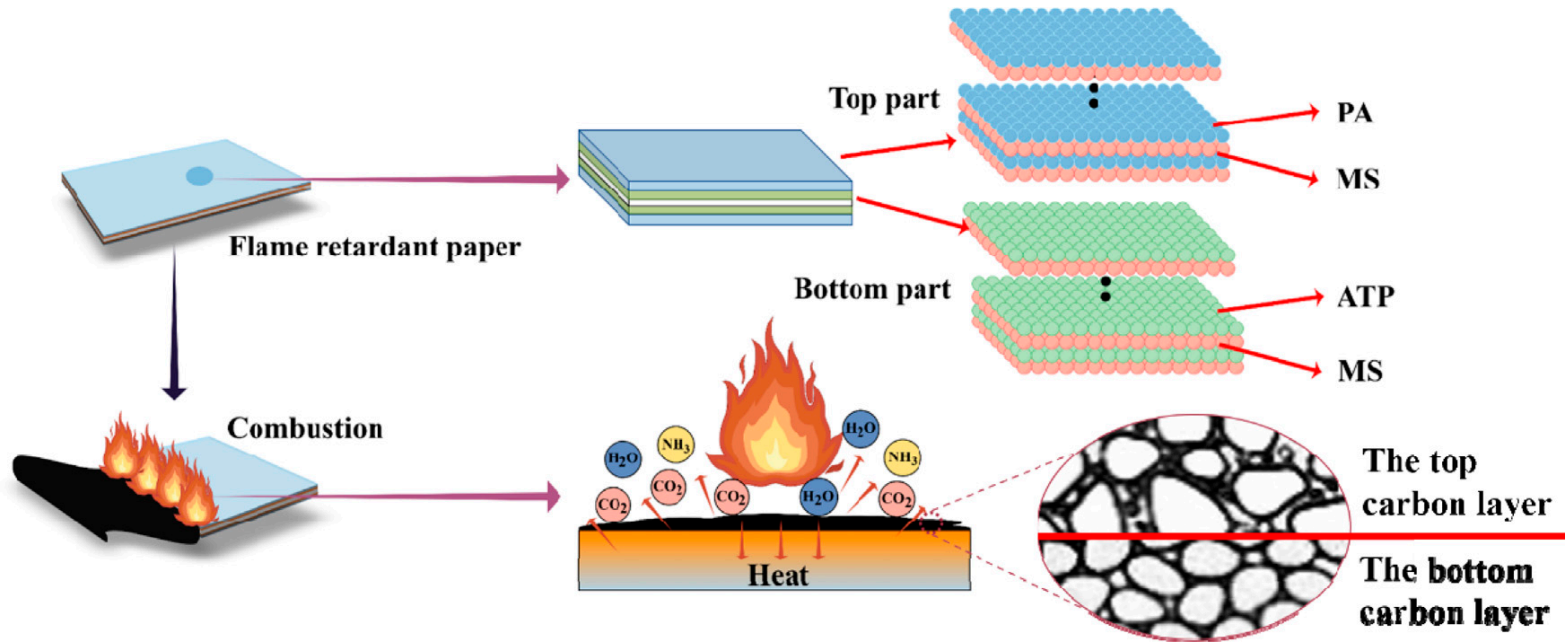
The flame retardant mechanism of bilayered coating (Drawn by Figdraw).

According to the intumescent flame retardant mechanism, the top and bottom parts of the bilayered coating have been designed with different thermal response temperatures. Figure 16 shows the flame retardant mechanism of bilayered coating. In the top part, PA will pyrolyze earlier when the temperature reaches 180°C–220°C, and then MS reacts to carbonize and generate char residues. This produces the top carbon layer and provides flame retardant protection to the treated paper. With the temperature continuously increasing, ATP in the bottom part gradually begins to pyrolyze when the temperature of the bottom part reaches 300°C–340°C. The pyrolysis of ATP leads MS in the bottom part to start carbonizing and generating the char residues underneath the carbon layer which has already been produced by the top part. Then the bottom carbon layer is formed and the flame retardant protection will be further improved. In the double-carbonized-intumescent process, the top carbon layer will become more loose due to the intumescent force from the bottom carbon layer, which enhances its heat insulation effect. In the same way, the bottom carbon layer will be more tight with the compressing force from the top carbon layer, which improves its ability to impede the penetration of flammable gases. Due to the property of the double-carbonized-intumescent, the bilayered flame retardant coating will provide more effective protection to the paper.

## 4 Conclusion

In this study, the fully bio-based bilayered flame retardant coating is employed to reduce the flammability of wood-base paper with the self-assembly method. According to the electrostatic self-assembly process, MS, ATP, and PA are deposited homogeneously on the paper surface to coat the cellulose fiber and fill the fiber gaps. When the paper is exposed to the fire, the flame retardant coating includes the bottom and top parts which will react successively with temperature increasing, and produce the superposed intumescent carbon layers that will provide good flame retardancy for paper. With the layers increasing, the LOI of paper samples performs better linear growth from 19.07% of 0 BL to 24.00% of 20 BL. Meanwhile, the burning rate quickly goes down and then keeps stable at about 200 mm/min after the coating reaches 8 layers.

During thermal exposure, the flame retardant coating reacts earlier than the paper and will generate more char residues by incomplete combustion. The carbon layer, constituted of char residues, will effectively reduce both peak and total heat release rates of paper. On mechanical properties, the coating improves the tensile strength and tearing resistance of paper, yet the folding endurance has slightly decreased. The water absorption of paper shows a reduced trend with coating layers increasing. The coated paper has a good biodegradation performance in the natural soil and water environments. The method presented in this study provides an environmentally sustainable approach for producing flame retardant wood-based paper.

## Data Availability

The original contributions presented in the study are included in the article/supplementary material, further inquiries can be directed to the corresponding authors.
